# The role of beta-adrenoreceptors in postoperative ileus in rats

**DOI:** 10.1007/s00210-023-02918-3

**Published:** 2023-12-29

**Authors:** Bitel Marcin, Sztormowska-Achranowicz Katarzyna, Kocić Ivan

**Affiliations:** Department of Pharmacology, Faculty of Medicine, Medical University of Gdańsk, Dębowa Str. 23, 80-204 Gdańsk, Poland

**Keywords:** Postoperative ileus, Rats, Propranolol, Metoprolol, ICI 118.551, SR58894A

## Abstract

The aim of the research was to evaluate the influence of antagonists of specific beta-adrenergic receptor subtypes on bowel motility following abdominal surgery in rat model of postoperative ileus. Bowel motility was measured by the intestinal transit of Evans blue introduced via orogastric tube after surgical procedures of skin incision, laparotomy and laparotomy with gut manipulation. Male rats were given individual adrenergic receptor subtypes antagonists intraperitoneally, and the influence of administered agents on intestinal transit of Evans blue was then evaluated. No statistically significant differences in the length of intestine in tested rats were observed. Propranolol administered prior to surgical procedure has shown protective effect on Evans blue migration in rats undergoing laparotomy and gut manipulation. Intestinal dye transit for propranolol doses of 10, 30 and 45 mg/kg was 18.00 ± 1.88c m, 23.75 ± 1.71 cm and 22.5 ± 2.43 cm, respectively, and for last two doses, statistically significant increase of dye passage was noted, compared to Evans blue transit of 11.00 ± 2.43 cm in the control group. No acceleration of dye migration was seen following administration of beta1-, beta2- and beta3-selective adrenergic receptor antagonist metoprolol, ICI 118.551 and SR58894A, respectively. Our research confirmed that propranolol at high doses, as seen by other researchers, improved bowel motility in early phase of postoperative ileus. That slight acceleration of intestinal dye transit after surgery with gut manipulation is rather connected with membrane-stabilizing action, than the receptor blocking effect, as this effect was not observed after the application of selective antagonists of respective subtypes of beta-adrenergic receptor.

## Introduction

Postoperative ileus (PI) is a common surgical complication, defined as transient bowel dysmotility following abdominal surgery with gut manipulation. Typical symptoms of PI include gas retention, bloating, nausea or vomiting and abdominal discomfort. The process is spontaneously reversible but leads to increased patient suffering, prolonged hospitalization and increased hospitalization costs, due to complications such as increased risk of aspiration pneumonia (Bauer and Boeckxstaens 2004; Namba et al. 2021). Typically in humans, dysmotility resolves the fastest in the stomach and the small intestine, followed by the large intestine, where it can last up to 72 h. Ileus lasting longer than 72 h after surgery is defined as paralytic ileus (Harnsberger et al. 2019). Current treatment techniques of PI involve mainly supportive measures.

Mechanisms underlying development of PI have not been fully explained. Both mechanical and humoral factors are related to PI pathogenesis, including neurogenic reflexes, inflammatory reaction in the intestines and pharmacological substances used during procedure (Mazzotta et al. 2020).

PI has two phases, early, lasting up to 3 h and late, up to 72 h. Administration of guanethidine and yohimbine during the surgical procedure or prior sympathectomy was shown to increase intestinal transit within 3 h after surgery (Fukuda et al. 2005).

Adrenergic reflexes are not the only cause of PI, as intestinal transit in control animals, not subjected to surgery, was greater than in animals subjected to surgery with prior adrenergic blockade.

It has been shown that mechanical stimulation of bowels in rats results in recruitment of macrophages residing in intestine wall, leading to release of various substances including nitric oxide and prostaglandins (Kalff et al. 1999). Additionally, the release of proinflammatory cytokines increases expression of adhesion molecules (P-selectin, intercellular adhesion molecule 1 (ICAM-1) and lymphocyte function-associated antigen 1 (LFA-1)) and leads to further leukocyte recruitment and inflammation (Kalff et al. 1999). Effects of prostaglandins and nitric oxide were also previously researched in our department (Korolkiewicz et al. 2003, 2004).

Furthermore, a link between adrenergic reflexes and inflammatory reaction in intestine wall has been shown. Delayed gastric emptying occurring after manipulation of intestine, followed by intestinal leukocyte recruitment was normalized by postoperative treatment with hexamethonium and guanethidine in mice. Increased expression of c-fos protein, marker of adrenergic activation in sensory neurons of spinal cord, has been reported (Kreiss et al. 2003).

As previously mentioned, the release of sympathetic mediators during invasive abdominal surgery has been described as one of mechanisms involved in PI pathogenesis.

The aim of this study was to research the influence of propranolol, which has previously shown beneficiary results in alleviating symptoms of PI in humans (Anderson et al. 1996; Hallerbäck et al. 1987) not supported by subsequent study (Ferraz et al. 2001), on intestinal transit in the in vivo model of PI in rats. Furthermore, we aimed to investigate which of the beta-receptor subtypes might be responsible for beneficiary effects of propranolol by pre-treating rats subjected to the same PI model with metoprolol, ICI 118.551 and SR58894A, a beta1-, beta2- and beta3-selective adrenoreceptor antagonist, respectively.

## Methods

Male Albino Wistar rats weighting 160 to 280g were kept in standardized conditions of temperature, moisture and lighting, fed with standard commercial rat chow. For 24 h prior to any procedures, rats were fasted with unlimited access to tap water. Procedures were performed by one researcher under diethyl ether anaesthesia. During the procedures, standard aseptic procedures were undertaken. Procedures were performed according to slightly modified protocol used by De Winter (1997) and in previous research in our department (Korolkiewicz et al. 2003, 2004).

The selected research model of postoperative ileus is based on the observation that the degree of inhibition of gastrointestinal motility depends on the strength and type of pain stimulus used during surgery. In preliminary phase of the research, we observed that skin incision (SI) reduced gastrointestinal transit by only approximately 9.3%, while laparotomy (L) and laparotomy followed with gut manipulation (L+M) resulted in significant decrease of Evans blue migration by approximately 46.1% and 79.3%, respectively, compared to untreated rats (UT). Due to the fact that the length of the surgical incision, especially of the muscle fascia and muscular tissue, was shown to influence gastrointestinal transit (Uemura et al. 2004), both the skin incision and laparotomy performed in this study had a standardized length of 3 cm. Manipulation was performed after gently removing intestines of the abdomen and placing them on sterile gauze. During the procedure, due to fragility of mesentery and mesentery vessels, the utmost care was taken to prevent excessive damage, as such damage affected motility recovery. Cases of visible ruptures were noted in eight animals, and these subjects were excluded from the study and statistical analysis.

In order to ensure standard treatment conditions, constant temperature of 38 ± 1 °C was maintained in the operating field using heat radiator, controlled by thermometer with a probe placed in the immediate vicinity of the operated animal. Intestines were kept moist using warm, sterile 0.9% sodium chloride solution to prevent drying.

In pilot series of experiments, rats were randomly divided into one of five groups. In first group (UT), which was control, untreated group, rats did not undergo any surgical procedures. In second group (E), ether anaesthesia was performed, with duration equal to subsequent groups (10 min). In three subsequent groups, rats undergone surgical procedures: standardized 3 cm skin incision (SI), 3 cm laparotomy (L) or 3 cm laparotomy followed by evisceration and gut manipulation (L+M).

Standardized gut manipulation was performed after gently pulling the intestine from abdominal cavity and laying it on wet sterile gauze. The gut was kept moist with sterile saline and standard temperature was maintained. Small intestine was then carefully massaged using fingers in sterile gloves; the procedure repeated 6 times within 6 min. Afterwards, the intestine was carefully reintroduced into abdominal cavity, and the wound closed with 3 sutures on the abdominal muscle and 3 sutures on the skin.

Subsequent procedures were performed after 1 h, time needed for the GI motility to recover from ether anaesthesia, according to prior research (De Winter et al. 1997). Rats were given 0.15 ml of Evans blue via orogastric tube.

The measurement of intestinal transit was evaluated by measurement of the distance from the pylorus to the farthest point of the Evans blue dye migration. Animals received 0.15 ml of dye 60 min after surgery via orogastric tube.

After exactly 30 min, animals were sacrificed by spine dissection, abdominal cavity reopened, intestines eviscerated and a clamp placed at the farthest point of dye migration. Small intestine was removed, and the distance from pylorus to the most distal point of Evans blue migration was measures. The total length of the small intestine was also noted.

In the second series of experiments, rats were divided randomly into subgroups, and the effects of increasing doses of propranolol, metoprolol, ICI 118.551 and SR58894A given intraperitoneally 2 h prior to surgical procedure on intestinal transit were studied in rats undergoing laparotomy with gut manipulation (L+M). Control animals (CG) were given an equal volume of 0.9% saline intraperitoneally 2 h prior to surgery.

In the third series of experiments, effects of propranolol was studied on intestinal transit in animals undergoing laparotomy (L) and 10-min diethyl ether anaesthesia (E). The size and symbols for all experimental groups are presented in Table 1.

**Table 1:** Classification and number of animals used in the experiment

Experimental stage	Group name	Group symbol (group size)
1st	Untreated	UT (*n* = 8)
	Ether anaesthesia	E (*n* = 8)
	Skin incision	SI (*n* = 8)
	Laparotomy	L (*n* = 11)
	Laparotomy and gut manipulation	L+M (*n* = 7)
2nd	Control group (0.9% NaCl)	CG (*n* = 6)
	Propranolol 10 mg/kg	P 10 (*n* = 10)
	Propranolol 30 mg/kg	P 30 (*n* = 12)
	Propranolol 45 mg/kg	P 45 (*n* = 6)
	Metoprolol 2 mg/kg	M 2 (*n* = 10)
	Metoprolol 10 mg/kg	M 10 (*n* = 10)
	Metoprolol 30 mg/kg	M 30 (*n* = 9)
	Metoprolol 100 mg/kg	M 100 (*n* = 8)
	ICI 118.551 0.3 mg/kg	ICI 0.3 (*n* = 10)
	ICI 118.551 1 mg/kg	ICI 1 (*n* = 9)
	ICI 118.551 3 mg/kg	ICI 3 (*n* = 10)
	ICI 118.551 10 mg/kg	ICI 10 (*n* = 10)
	SR58894A 1 mg/kg	SR 1 (*n* = 5)
	SR58894A 3 mg/kg	SR 3 (*n* = 5)
	SR58894A 10mg/kg	SR 10 (*n* = 4)
3rd	Ether anaesthesia	E (*n* = 8)
	Ether anaesthesia and propranolol 30 mg/kg	E + P 30 (*n* = 5)
	Laparotomy	L (*n* = 11)
	Laparotomy and propranolol 30 mg/kg	L + P 30 (*n* = 5)

### Drugs

Propranolol, metoprolol, ICI 118.551 and SR 58894A were purchased from Sigma-Aldrich, diethyl ether was purchased from Polish Chemical Reagents, Kutno and 0.9% saline was purchased from Frasenius Kabi, Kutno. Evans blue was purchased from Sigma-Aldrich and prepared by dissolving 50 mg of dye in 1 ml of 0.9% saline.

### Statistics

During the study, the total length of the test animal’s intestine from the pylorus to the cecum and the distance from the pylorus to the farthest point of dye penetration were measured. Therefore, statistical analysis results are presented as centimeters from the pylorus to the farthest point of the Evans blue intestinal transit in centimeters ± standard error of the mean (SEM). Results were analyzed using StatSoft, Inc. (2011) STATISTICA, version 10 with on-site license for Gdansk Medical University. Results were compared using one-way analysis of variance (ANOVA) and post-ANOVA Bonferroni test. *P* values of less than 0.05 were assumed to indicate statistically significant difference.

## Results

### Total length of the small intestine

**Fig. 1:**
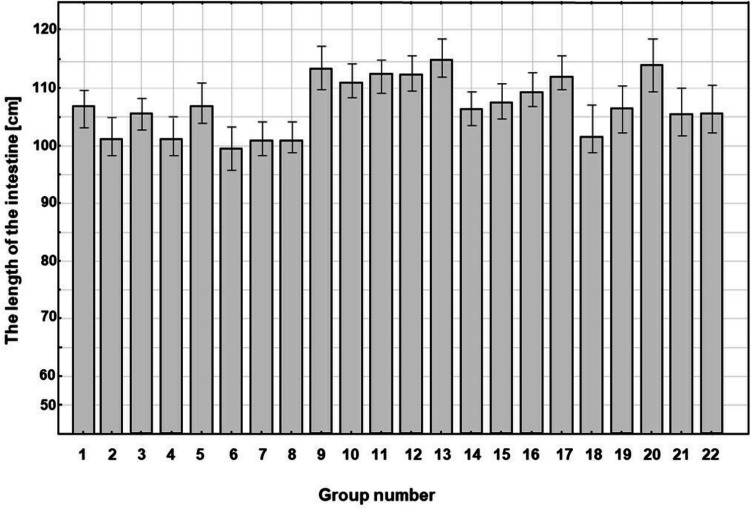
The length of the small intestine was measured from the pylorus to the caecum in all groups of rats used in the experiments. The results in the groups *n* = 4–12 are presented as the length of the intestine in cm ± standard error (SEM). Using the one-way ANOVA test and Bonferroni post-hoc test, no statistically significant differences were found between the groups. Legends for the Fig. 1 (group number, group name) are as follows: 1, untreated; 2, ether anaesthesia; 3, skin incision; 4, laparotomy; 5, laparotomy and gut manipulation; 6, control group with 0.9% NaCl; 7, propranolol 10 mg/kg; 8, propranolol 30 mg/kg; 9, propranolol 45 mg/kg; 10, metoprolol 2 mg/kg; 11, metoprolol 10 mg/kg; 12, metoprolol 30 mg/kg; 13, metoprolol 100 mg/kg; 14, ICI 118551 0.3 mg/kg; 15, ICI 118551 1 mg/kg; 16, ICI 118551 3 mg/kg; 17, ICI 118551 10 mg/kg; 18, SR59230A 1 mg/kg; 19, SR59230A 3 mg/kg; 20, SR59230A 10 mg/kg; 21, laparotomy and propranolol 30 mg/kg; 22, ether anaesthesia and propranolol 30 mg/kg

No significant differences were discovered in total length of the small intestine among all groups of tested animals; therefore, results were presented by the main distance of the most distal Evans blue migration from pylorus in cm ± standard error of the mean (SEM) in each tested group (Fig. 1).

### The effect of diethyl ether and surgical procedures on intestinal transit

**Fig. 2:**
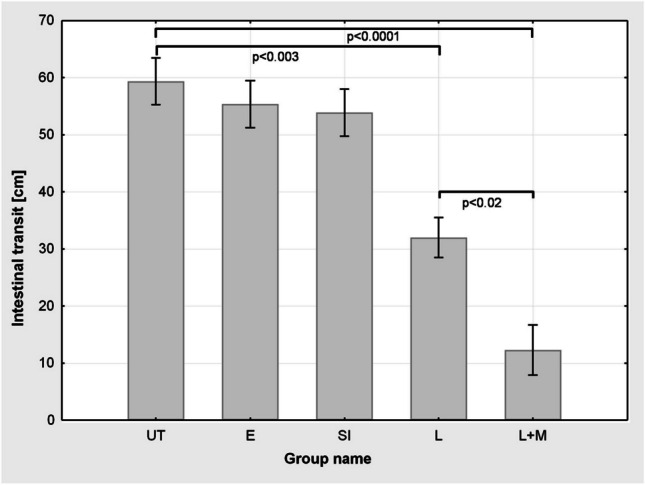
The obtained values of the intestinal transit of the dye in the preliminary experiments in the group not subjected to any procedures (UT) and groups subjected to the following: ether anaesthesia (E), skin incision (SI), laparotomy (L) and laparotomy with bowel massage (L+M). Results are shown as cm passage of Evans dye in the intestine from the pylorus ± standard error (SEM). Statistically significant differences between the groups with the size of *n* = 7–11 were obtained: UT, E and SI vs L (*p* <0.003), UT, E and SI vs L+M (*p* < 0.0001), and also between L vs L+M (*p* < 0.02)

Evans blue migrated in the small intestine over 59.37 ± 4.10 cm in the untreated group (UT). Diethyl ether anaesthesia (E) and skin incision (SI) did not significantly affect intestinal transit of the dye, which was 55.37 ± 4.10 cm in the former and 53.87 ± 4.10 cm in the latter group. Both laparotomy (L) and laparotomy followed by gut manipulation (L+M) significantly reduced intestinal motility with Evans blue being transferred to 32.00 ± 3.50 cm and 12.28 ± 4.38 cm, respectively (Fig. 2).

### The effect of beta-adrenoreceptor antagonist administration on intestinal transit after laparotomy and gut manipulation

**Fig. 3:**
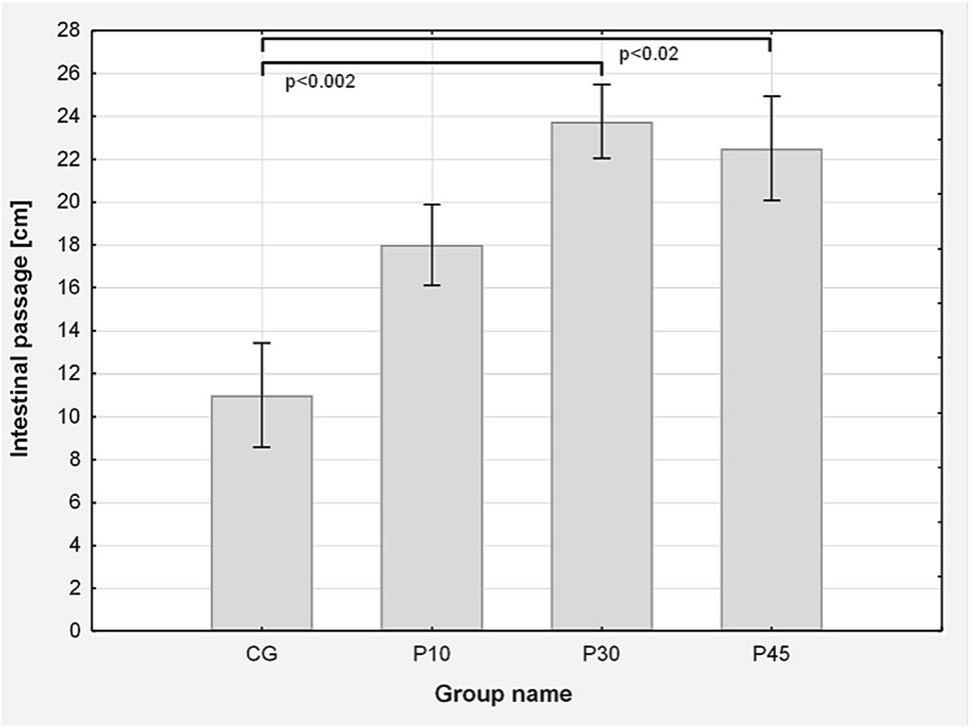
Effect of propranolol in doses of 10, 30 and 45 mg/kg B.W. (P10; P30; P45 respectively) on the intestinal transit of the dye in the laparotomy and intestinal massage group compared to the control (CG) group. Results are shown as cm passage of Evans dye in the intestine from the pylorus ± standard error (SEM). Statistically significant differences between the groups with the size of *n* = 6–12 were obtained: P30 vs CG (*p* < 0.002) and P45 vs CG (*p* < 0.02)

In control group (CG) which received 0.9% saline intraperitoneally 2 h prior to surgery, intestinal transit of the dye after laparotomy followed by gut manipulation was found to be 11.0 ± 2.43 cm. Propranolol in doses 30 and 45 mg/kg (P30 and P45) significantly reversed the inhibitory effects of laparotomy and gut manipulation compared to CG group rats, with intestinal transit, respectively, 23.75 ± 1.71 cm and 22.5 ± 2.43 cm. Propranolol in the dose of 10 mg/kg did not manifest significant improvement of intestinal transit of dye compared to CG group (Fig. 3). Administration of propranolol in the dose of 60 and 100 mg/kg resulted in premature death of test animals.

**Fig. 4:**
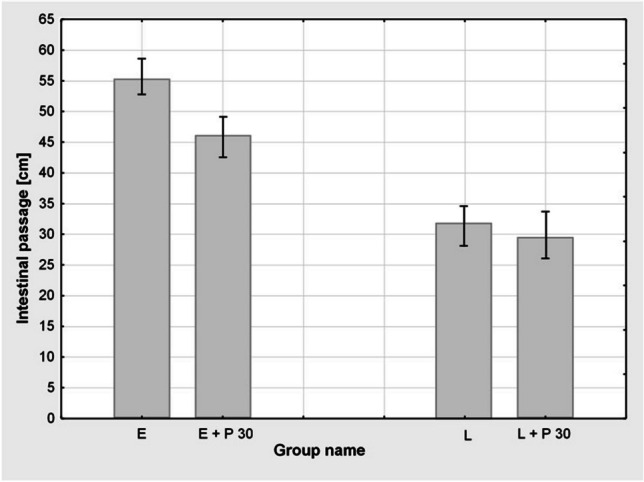
Effect of administration of propranolol at a dose of 30 mg/kg B.W. in rats subjected to ether anaesthesia (E+P 30) or laparotomy (L+P 30) compared to groups which underwent only ether anaesthesia (E) or laparotomy (L), respectively. *p* results are presented in cm of passage of Evans dye in the intestine from the pylorus ± standard error (SEM). The group size was *n* = 5–11. The administration of the drug did not cause a statistically significant increase in dye passage after anaesthesia with ether or laparotomy at the assumed test probability level of *p* < 0.05

Propranolol administered in the optimal dose of 30 mg/kg did not manifest significant effects on dye migration in rats subjected only to laparotomy (L+P30) or diethyl ether anaesthesia (E+P30) compared to animals subjected to those procedures and not given the drug (Fig. 4).

**Fig. 5:**
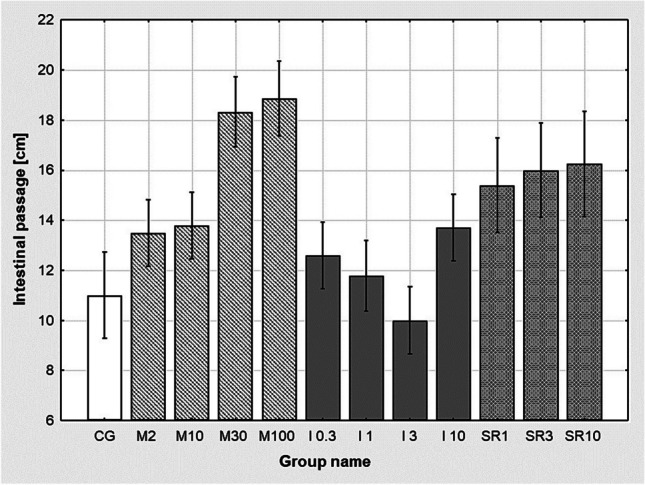
Effect of metoprolol in doses of 2–100 mg/kg B.W. (M 2; M 10; M 30; M 100), ICI 118.551 in doses of 0.3–10 mg/kg B.W. (ICI 0.3; ICI 1; ICI 3; ICI 10), SR 58894A in doses 1–10 mg/kg B.W. (SR 1; SR 3; SR 10) on the intestinal passage of Evans’ dye in the laparotomy and bowel massage group compared to the control group (CG). There were no statistically significant differences between the groups of *n* = 4–10 at the assumed level of the test probability *p* < 0.05

There were no significant differences in Evans blue migration in any groups pre-treated with metoprolol 2–100 mg/kg B.W. (M2–M100), ICI 118.551 0.3–10 mg/kg B.W. (I0.3–I10), SR 58894A 1–10 mg/kg B.W. (SR1–SR10) compared to control group (CG) (Fig. 5).

## Discussion

The role of the sympathetic nervous system in the regulation of intestinal motility has already been the subject of many studies. This paper analyzes the contribution of specific adrenergic receptor types and subtypes in the biological model of postoperative ileus in rats.

The methodology used in this work is in line with good laboratory practice and has been used in prior research (Ciechanownicz et al. 2006; De Winter et al. 1997; Korolkiewicz et al. 2003). The animals used for experiments were treated in a humane manner, under general anaesthesia with diethyl ether with the effects on gastrointestinal peristalsis in rats limited to 1 h **(**Bueno et al. 1978; De Winter 1997). The disadvantage of diethyl ether anaesthesia is the difficulty of standardizing the dose. An overdose leads to inhibition of the medulla, causing circulatory collapse and respiratory arrest. The depth of anaesthesia was thus assessed based on the response to the pain stimulus and the depth of breath of test animals. Among all tested subjects, five lethal cases of ether overdose occurred in the course of this study and those were excluded from the statistical analysis.

Slow induction compared to isoflurane or even halothane is another disadvantage of ether anaesthesia. This can lead initially to animal’s arousal, before proper surgical anaesthesia is achieved. We found that the duration of the arousal can be shortened by rapid increase of concentration of diethyl ether in the breathing mixture. To standardize the exposure to diethyl ether, the duration of anaesthesia was the same for all groups, lasting 10 min, regardless of the type of surgical procedure performed.

Important disadvantages hindering practical application of the results include inter-species differences: in humans, the colon is the main part affected by postoperative ileus, while in rats, postoperative propulsion is mostly reduced seen in the small intestine (Holte and Kehlet 2002). In addition, the chosen model only evaluated the early phase of postoperative ileus in rats, leaving the delayed phase of the phenomenon untested.

Another limitation of direct measurement of Evans blue given via orogastric tube is that it reflects not only the intestinal transit but also the stomach emptying. On the other hand, results obtained in the preparatory phase of this study confirmed previous reports showing reproducibility and correlation of dye migration reduction with the degree of invasiveness of surgical procedures (De Winter et al. 1997; Korolkiewicz et al. 2003, 2004). Therefore, we concluded that the chosen method of measuring the gastrointestinal passage, despite its setbacks, satisfactorily reflects the early phase of postoperative ileus in rats.

In the following part of the experiments, the effects of the beta-adrenoreceptor antagonists were evaluated. The intraperitoneal route of administration was chosen over intramuscular due to volume of the drug solution and larger surface of absorption. Intravenous route was not suitable for beta1-antagonists due to hypotension and severe bradycardia upon drug administration. The main result of our study is that selective beta1 and beta2 receptor antagonists had no significant effects on intestine motility, while propranolol at 30 and 45 mg/kg B.W. increased it. Metoprolol at a maximal tolerated dose 100 mg/kg B.W. had similar but not significant effect (*p* = 0.0055).

Propranolol is a nonselective beta-adrenoceptor antagonist reported to have membrane-stabilizing properties, but it does not own intrinsic sympathomimetic activity (Al-Majed et al. 2017). In the literature, one can find conflicting information about the effect of propranolol on intestinal passage in postoperative intestinal obstruction in rats. In a study conducted in patients undergoing colon resection, it was found that intravenous administration of propranolol shortens the time to stool occurrence (Hallerbäck et al. 1987). Another study found that oral administration of propranolol started before and continued after surgery does not shorten the duration of obstruction and has no effect on the electrical activity of the colon (Ferraz et al. 2001). In both cases, doses much lower than those used in this study were administered.

To attempt to explain the effect of propranolol on accelerating passage in the model of postoperative intestinal obstruction used, its additional pharmacodynamic properties should be considered. This drug is not only a model antagonist of beta1- and beta2-adrenergic receptors. At high doses, it is attributed a cell membrane stabilizing action similar to quinidine or lidocaine. It has been established that this effect is independent of adrenergic receptor blockade, as it also occurs after the use of the d-propranolol stereoisomer, which has no affinity for beta receptors. Moreover, this effect was not observed after the use of metoprolol. It has been established that this action is based on inhibiting voltage-dependent sodium channels (Wang et al. 2010). The action on sodium channels can cause a local anesthetic effect. In addition, in vitro studies have observed the antioxidant action of propranolol used at high doses. This action consisted in reducing the production of oxygen radicals associated with the neutralization by propranolol of the action of pro-inflammatory cytokines such as phospholipids, LPC, PAF or LPAF on the cell membrane of human neutrophils. As in the case of the previous cited work, in this study, both stereoisomers of propranolol were observed to be equally effective, and this effect was not observed after the use of other beta-blockers, including metoprolol (Anderson et al. 1996). Other authors observed the presence of beta2-adrenergic receptors on the surface of macrophages. After using propranolol, a decrease in free oxygen radical production was observed in macrophages, which was associated with blocking beta2-adrenergic receptors by this drug. However, no selective antagonists of beta-adrenergic receptors were used in this study, which does not allow to confirm that it is precisely antagonism towards the beta-2 adrenergic receptor and not cell membrane stabilizing action that is responsible for this effect (Shen et al. 1994). Moreover, attention should be paid to a previously mentioned controlled prospective study conducted on humans where acceptable doses of propranolol ranging from 80 to 160 mg per day were evaluated, and no statistically significant difference was found in shortening symptoms of postoperative intestinal obstruction, as well as directly measured intestinal electrical activity after the use of propranolol compared to placebo (Farraz 2001).

## Conclusions

Based on the results obtained in this work, it is not possible to unequivocally determine the mechanism of accelerating passage after premedication with propranolol. The observation that the effect of drug action appears at relatively high doses and lack of effect on intestinal passage after administration of selective antagonists of adrenergic receptors beta1, 2 and 3 allows to put forward a hypothesis that observed acceleration of passage may be associated with cell membrane–stabilizing action of propranolol causing local anesthetic effect and antioxidant effect on neutrophils.

Unfortunately, the doses of propranolol necessary to induce this effect are so large that if they were used in humans, there would be severe side effects from the cardiovascular system such as hypotension, bradycardia or heart conduction system blockage. This limits or even makes it impossible to practically apply propranolol at such doses in postoperative intestinal obstruction in humans.

## Data Availability

Not applicable
